# Transporting: What Is It and How Do You Do It?

**DOI:** 10.1007/s40471-025-00374-6

**Published:** 2025-10-18

**Authors:** Michael Webster-Clark, Alexander Breskin, Emilie D. Duchesneau, Kara E. Rudolph

**Affiliations:** 1 Wake Forest University School of Medicine, Winston-Salem, NC, US; 2 McGill University Department of Medicine, Montreal, QC, Canada; 3 University of North Carolina at Chapel Hill, Chapel Hill, NC, US; 4 Regeneron Pharmaceuticals Inc, Tarrytown, NY, US; 5 Mailman School of Public Health, Columbia, New York, NY, US

**Keywords:** External validity, Transportability, Generalizability, Weighting, Outcome modeling, Breast cancer

## Abstract

**Purpose of Review:**

Transportability, one of the twin faces of external validity (alongside generalizability), refers to the ability to use effect estimates in a study population to understand effects in a different population. In this review, we aimed to provide an overview of ongoing methodological developments in the field of transportability and provide a tutorial walking through key steps in the transportability process.

**Recent Findings:**

We cover recent work done to distinguish the concept of transportability from generalizability (or external validity more broadly), define core conditions necessary for transporting treatment effects to a different target population, outline approaches to identify sufficient adjustment sets, and design estimators to estimate transported treatment effects. We then illustrate the application of these methods through a case study comparing the effects of two adjuvant chemotherapies for breast cancer in patients within the National Cancer Database, a large national cancer registry, using effect estimates transported from a randomized controlled trial.

**Summary:**

While external validity, generalizability, and transportability have long been recognized as important elements of epidemiology, they have historically been treated interchangeably and discussed qualitatively in discussion sections of manuscripts. Over the past two decades, however, major strides have been made to formally define these concepts and introduce analytic methods for them that are valid under well-defined conditions.

## Introduction

External validity – the extent to which findings within a study sample can be used to draw inferences about a population of interest (i.e., a target population) – is recognized as an important aspect of epidemiology and public health [1–4]. Sometimes, we are interested in if the estimates from a study sample will apply to a separate population that is partially or completely distinct from the source population, a concept known as transportability [5, 6]. Other times, we are unsure whether estimates from a study sample accurately reflect the wider population that the study was sampled from, a concept known as generalizability [7, 8]. A lack of external validity has long been recognized as a potential limitation of randomized trials and observational studies [9–11]. In the past, researchers qualitatively described whether specific results applied to a possibly unstated target population [12, 13] formally extrapolated absolute effect estimates to other populations while assuming ratio-scale effects were constant [14, 15]or evaluated proportions of patients in the real-world qualifying for trials [16, 17].

The potential outcomes framework [18] and directed acyclic graphs (DAGs) [19–21] have provided ways to reason about (and solve, under well-defined conditions) threats to internal validity like confounding, selection, and information bias. At the end of the 2000 s, people began exploring ways to adapt analytic tools developed to solve internal validity problems to external validity problems [22, 23] These tools include inverse probability weighting to generalize estimates [8] inverse odds weighting to transport estimates [24], outcome modeling and standardization [25], and doubly robust analyses [25, 26]. The underlying assumptions for transporting treatment effects were formalized [23, 27, 28] and graphs or diagrams were adapted to facilitate, and identify adjustment sets for, generalizing and transporting [27, 29, 30].

The rapid development of methods to conceptualize external validity, generalizability, and transportability has introduced both opportunities and challenges, with definitions and best practices constantly evolving. Here, we define transportability, outline research questions it addresses, discuss strategies for variable selection, and describe different estimators for transportability. We then illustrate these methods using a simple case study transporting effects from a randomized controlled trial to an external real-world target population. Finally, we highlight ongoing developments in the field.

## Formally Distinguishing Generalizability and Transportability

In the past, “generalizability” and “generalizing” were catch-all terms for issues arising from differences between a study population and a target population of interest, regardless of the relationship between the populations. For example, the 1988 New England Journal of Medicine publication of preliminary results from the Physicians Health Study (PHS)—a randomized trial assessing the effect of aspirin on cardiovascular mortality—includes the following in its discussion [13]:

“The extraordinarily low cardiovascular mortality rate among participants in this trial raises the question of the generalizability of its findings. Specifically, it is necessary to consider whether the beneficial effect of aspirin on fatal and nonfatal myocardial infarction found in this population would also apply to the physicians who were unwilling or ineligible to participate, as well as to U.S. men who are not physicians.”

In other words, the authors identify estimating effects in two targets as “generalizability” questions. The first, all trial-eligible U.S. physicians, is a population the study was sampled from and is nested within. The second, all U.S. men who are not physicians, is disjoint from the study population.

**Fig. 1:**
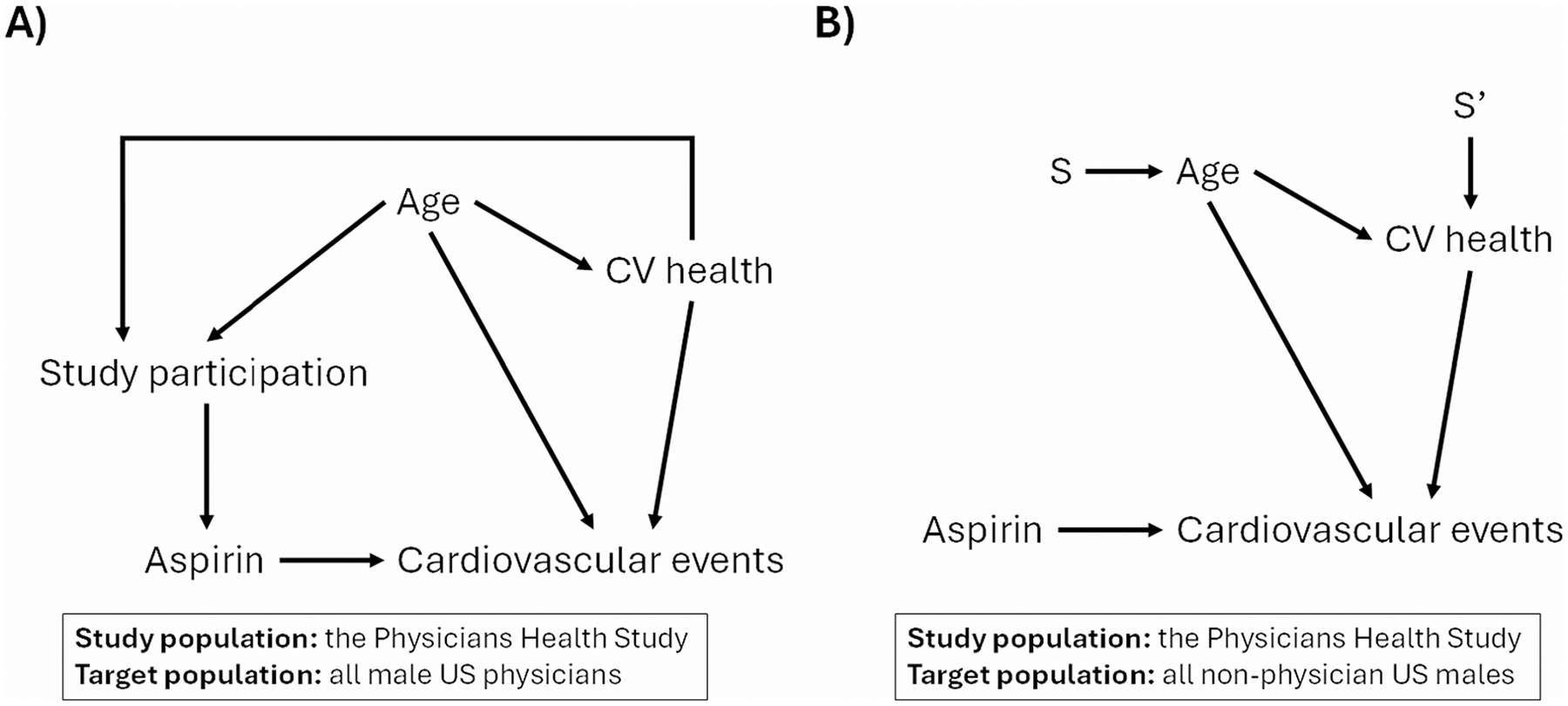
Directed acyclic graph showing possible causal relationships between cardiovascular health, age, study participation, aspirin use, cardiovascular events when selecting physicians to participate in the Physicians Health Study (panel **A**) and selection diagram indicating possible causal shift between the Physicians Health Study and the non-nested target population of all non-physicians US males (panel **B**)

This creates some important theoretical differences between those two target populations [31]. For example, one could create a DAG (Fig. 1 A) describing the relationship between age, cardiovascular health, and participation in the PHS among all men that are U.S. physicians (whether they were included in the trial or not) [29]. The edges in the DAG are causal relationships, meaning that if we were to intervene on these baseline characteristics in the population of male physicians in the U.S., it would alter their probability of inclusion in the study sample. Once we condition on study participation, there are no open backdoor paths from treatment to the outcome within the study sample. When creating an analogous DAG for the target population of men who are not U.S. physicians, we must address the fact that none of these people are sampled into the study and no intervention (aside from making them physicians) would impact their probability of study inclusion. A more theoretically accurate way to capture the differences between the two populations is with a selection diagram [5] (Fig. 1B). In these diagrams, S (and S’) nodes are drawn with “arrows” into nodes on the graph to represent variables where either the distribution of the variable (e.g., the age distribution of non-physician US males differs from the age distribution of male US physicians) or the relationships between other variables and the distribution of the variable (e.g., the effect of age on CV health is different for non-physician US males and male US physicians) differs. These arrows usually go from S to the nodes whose distributions differ across populations; unlike DAG arrows, these do not represent causal effects.

**Fig. 2:**
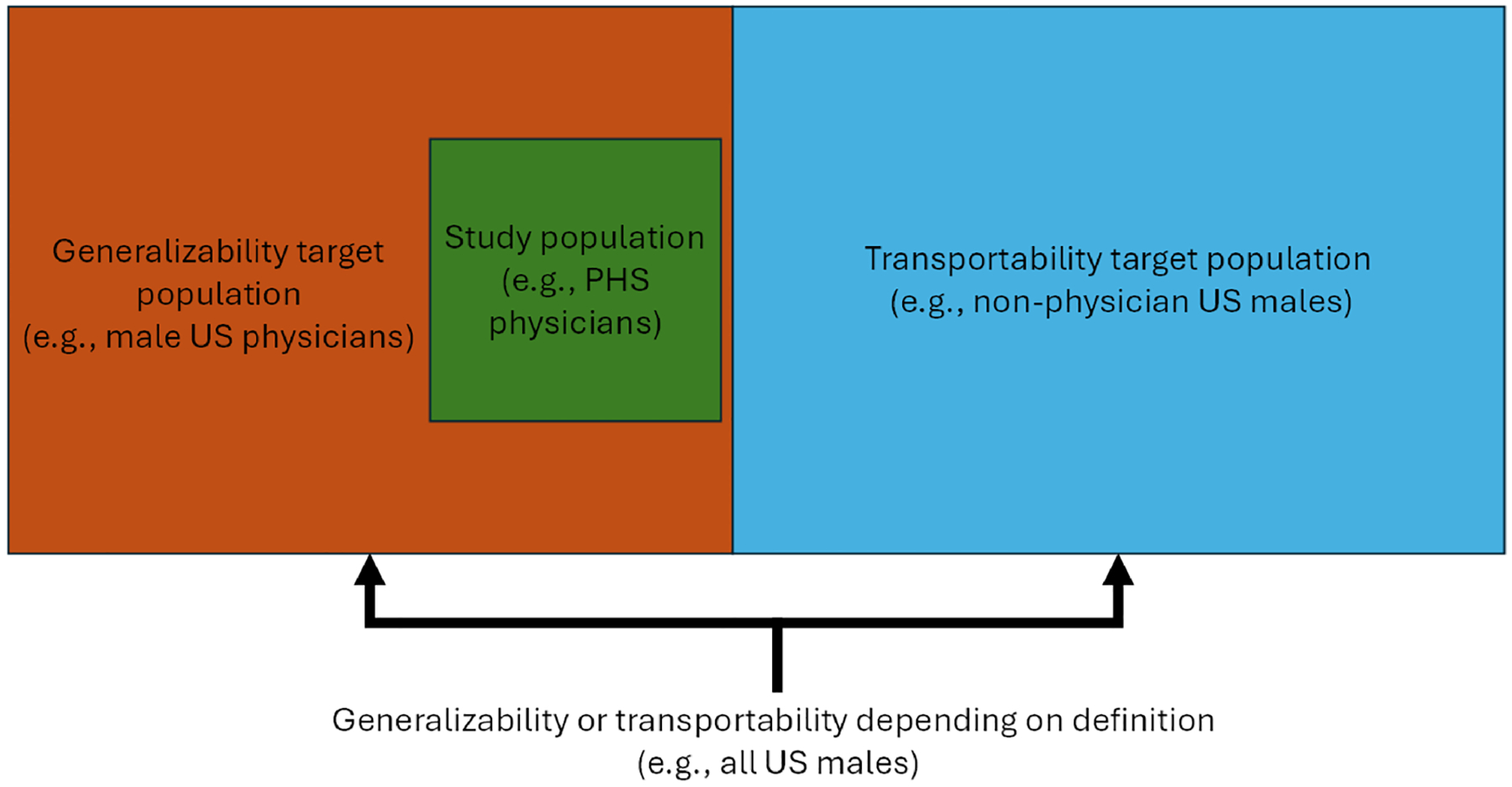
Visual representation of how “nesting” of populations impacts whether a study involves generalizability or transportability

Both situations involve external validity and potential type 2 selection bias [32], with “type 2” referring to the bias arising from the processes that “selected” the study population from the target population rather than a “type 1” bias arising from “selecting” the analytic sample from the study sample. They differ in how the study population relates to and is selected from the target population. When the study population is entirely sampled and actually selected from the target population (e.g., the wider population of U.S. physicians that are men), the issue concerns generalizability. When the target population is at least partially disjoint from the study population (e.g., U.S. men who are not physicians) and some individuals could not have been selected, it is a matter of transportability [4]. Sometimes, this distinction is conceptualized via figures like Fig. 2.

Though generalizing and transporting involve conceptual differences when reasoning about how study and target populations differ, analytic approaches to address each are analogous. In both settings, data from a source population is used to extend inferences to a target population. For the sake of standardized terminology and notation going forward, we will be operating under the assumption we are transporting effect estimates from a study population to a target population of separate individuals.

## Transportability Conditions and Variable Selection

Suppose we are interested in using data from a study to estimate the effect of a binary treatment *A* on an outcome *Y* in a separate target population. The study comprises *N*_*S*_ individuals indexed by *i* ∈ 1, …*, N*_*S*_, and the target population comprises *N*_*T*_ individuals indexed by *i ∈ N*_*S*_ + 1, …*, N*_*S*_ + *N*_*T*_. Let *S*_*i*_ = 1 if person *i* is included in the study, 0 otherwise. Additionally, some set of covariates, *W*, is collected in both populations. The observed data for an individual is *O*_*i*_ = {*S*_*i*_*, W*_*i*_*, S*_*i*_*A*_*i*_*, S*_*i*_*Y*_*i*_}. The transported average treatment effect (tATE) is defined as *E*(*Y*_1_*−Y*_0_*|S*=0), where *Y*_1_ and *Y*_0_ represent the potential outcomes under treatments *A* = 1 and *A* = 0, respectively. To identify the tATE from the observed data and in the absence of conditional exchangeability in the target population [28], several assumptions must be satisfied.

First, the study population must satisfy fundamental causal identifiability conditions sufficient for internal validity. These may include consistency (i.e., that *Y*_*a,i*_ = *Y*_*i*_ if *A*_*i*_ = *a*, or treatment variation irrelevance plus no interference) [33], conditional exchangeability in the study population (i.e., that *Y*_*a*_ ⊥ *A|W, S*=1 for *a* ∈ 0, 1, the absence of unmeasured confounding) [34], and positivity (i.e., that Pr(*A =a|W* = *w, S* = 1) > 0 if Pr(*W* = *w|S* = 1) > 0 for *a* ∈ 0,1, meaning all individuals have a non-zero probability of receiving either treatment) [35].

In addition to internal validity, transportability requires additional conditions to achieve external validity for the transported ATE, sometimes referred to as *S*-admissability [23, 28], *S*-admissability, *E*(*Y*_*a*_*|W, S* = 1) = *E*(*Y*_*a*_*|W, S* = 0) for *a* ∈ 0, 1, means that conditional on the variable set *W* the distribution of potential outcomes under each level of treatment is the same in the study and target populations. Importantly, this *W* may not be the same *W* required for internal validity because one relates to study selection and one, both, or neither process has the potential to be randomized. In other words, this condition focuses on exchangeability of outcomes between study and target individuals, rather than exploring exchangeability of outcomes between arms of the study. Note that if there is conditional exchangeability within the target population, an alternative type of *S*-admissability takes the form of transportability of the outcome model to allow fusion of data from the trial and target populations [28].

We can break *S*-admissability down into distinct components, analogous to the conditions required for internal validity, with a focus on the difference scale and effect measure modification [23]. First, external consistency requires that every individual i would experience the same outcome under a given level of treatment regardless of whether they were in the study or target, *Y*_*a,s*_ = *Y*_*a*_ for *a* ∈ 0,1, *s ∈* 0,1, where *Y*_*a,s*_ is the potential outcome in which a person receives treatment a and is in population s. This implies participation in the study does not directly impact outcomes under a given level of treatment and that the treatment under consideration is identical in both settings (or that we are considering extending the exact version applied in the study in the target). Second, external conditional exchangeability requires a sufficient set of measured variables that eliminates potential type 2 selection bias (see the following section on implications for variable selection). Finally, external positivity requires any covariate combinations of *W* necessary for conditional exchangeability that occur in the target population (*S* = 0) also occur in the study population (*S* = 1); Pr(*S* = 0*|W* = *w*)>0 *if* Pr(*W* = *w|S* = 1) > 0. Unlike internal positivity, external positivity is one-directional; there is no requirement for covariate combinations present only in those with *S* = 0 to be present in those with *S* = 1.

### Implications for Variable Selection

The conditions for transportability we describe have important implications for variable selection. One option to identify a sufficient adjustment set is to block all paths from selection (S) nodes to the outcome in a selection diagram [5]. However, this adjustment set may not be minimally sufficient, as it may include variables that do not threaten transportability (e.g., variables that do not modify the effect on the particular scale of interest) and potentially inflate the variance of effect estimates [28, 36]. It also may result in the inclusion of unnecessary mediators, complicating analyses and introducing additional assumptions about the transportability of mediated effects.

Specifying an effect measure scale (e.g., additive) can result in smaller minimally sufficient adjustment sets with improved precision vs. adjustment sets obtained when blocking all paths from selection nodes to the outcome. When transporting the risk difference, for example, the transport step need only adjust for variables that are risk difference effect modifiers and differ in distribution between the source and target populations [28, 37]. Variable selection based on effect measure modification cannot be done using DAGs or selection diagrams alone [38–40] and requires knowledge around marginal and conditional effect measure modification that may not be available [41, 42]. Moreover, variables that are not, strictly speaking, effect measure modifiers may be necessary to transport some effect measures (particularly non-collapsible effect measures such as the odds or hazards ratios) [37]. Mediators of the treatment-outcome association may also be required to transport estimates even when they are not “traditional” effect measure modifiers, necessitating more complex statistical models and introducing additional assumptions [28]. In practice, variable choice when transporting often reflects pragmatic concerns about sample size and data availability.

## Analytic Approaches

Once the adjustment set has been identified and the variables included in it harmonized, several analytic approaches exist for transporting treatment effect estimates. These approaches are related to common confounding control methods and include weighting, outcome modeling and standardization, and doubly robust approaches such as augmented inverse odds weighting (AIOW) and targeted maximum likelihood estimation (TMLE) [4, 6, 7, 25, 28] Strengths of each approach are listed in Table 1.

### Inverse Odds Weighting (IOW)

Weighting is a class of estimators where individuals within a population receive weights creating a “pseudopopulation” with the same covariate distribution as a target population [2, 28]. IOW is a variation employed when the trial and target are completely separate populations, as in our transportability context [24, 31]. To construct IOW, a model is fit for the probability of membership in the study sample conditional on the adjustment set. This can be done using parametric regression or any other well-behaved prediction approach. Each participant is assigned a weight equal to their covariate-conditional inverse odds of membership in the study population. The weighted population has the same distribution of covariates in the adjustment set as the target population. This is analogous to applying standardized mortality ratio (SMR) weights to an untreated population to generate a weighted population with the same covariate distribution as the treated population [31]. If there is significant informative attrition or censoring after the index date, these weights should be combined with inverse probability of censoring weights (IPCW) to avoid selection bias [43].

Unfortunately, if covariates in the adjustment set are imbalanced between the treatment groups in the study population whether due to confounding or random chance, that imbalance can increase after weighting [44]. Suppose 40% of one treatment group in the study is female, and 30% of the other treatment group is female, and the target population is 70% female. If the treatment groups are of equal size, and we fit IOW to the target population, the 40% vs. 30% imbalance would become an 80% vs. 60% imbalance, worsening confounding. To avoid this, researchers can create separate IOW for each treatment regimen within the study or fit inverse probability of treatment weights in the trial and combine them with overall IOW.

### Outcome Modeling with Standardization

Outcome modeling with standardization (sometimes referred to as the g-formula) [45, 46] focuses on modeling associations between treatment, the adjustment set, and the outcome, rather than associations between the adjustment set and study membership. Transporting estimates with this approach involves using the study sample to construct a model predicting potential outcomes under each treatment regimen of interest conditional on adjustment variables. From this model, predicted outcomes are generated for each member of the target and results are averaged to produce a final estimate. Importantly, the model must generate accurate predictions for the target population, based on its covariate distribution, under each treatment of interest. Contrasting these predictions, in turn, yields the final transported ATE.

### Doubly Robust Estimators

One straightforward doubly robust estimator is augmented inverse odds weighting (AIOW) [4]. It is unbiased if the study membership, treatment, and censoring models or the outcome model are correctly specified. AIOW fuses the outcome modeling with odds weighting, with outcome predictions for the target population “corrected” based on the model’s performance within the study sample weighted to resemble the target population (making sure to appropriately address loss-to-follow-up). Notably, due to favorable statistical properties, AIOW allows the study membership and censoring model or the outcome model to be specified with flexible machine learning methods, including SuperLearner [4], reducing the likelihood of model misspecification. Further, it is semiparametric efficient [47], meaning it is the most precise estimator in its class.

Targeted maximum likelihood estimation [26] is an alternative doubly-robust estimator. Like AIOW, TMLE will be unbiased if the outcome or study membership, treatment, and censoring models are specified properly. TMLE uses predictions from the study membership, treatment, and censoring models to update predictions from the outcome model such that they target the parameter of interest. The strengths of AIOW are shared by TMLE, including incorporating SuperLearner and flexible machine learning methods, but unlike AIOW, TMLE is guaranteed to respect the natural bounds of the parameter of interest (e.g., −1 to 1 for a risk difference) yielding finite sample advantages.

## Case Study Transporting Treatment Effects to A real-world Target Population of Patients with Breast Cancer

### Trial Data

As a demonstration of IOW and outcome modeling and standardization, we obtained individual-level data on 3,871 patients participating in a multi-center randomized trial comparing disease-free survival under two adjuvant (i.e., post-surgical) chemotherapy regimens (doxorubicin with cyclophosphamide, AC vs. paclitaxel, T) [48]. The trial (NCT: 00041119) included women with breast cancer with zero to three positive axillary nodes and enrolled patients between 2002 and 2010 from across the United States. Trial data were obtained from Project Datasphere^™^.

### Target Population Data

We used the National Cancer Database (NCDB) Participant Use Files [49], a national hospital-based cancer registry of incident cancer cases, to identify a target population of 46,739 women diagnosed with breast cancer with zero to three positive axillary nodes between 2018 and 2022 who underwent surgery within 6 months of diagnosis with or without chemotherapy. As the trial population (patients diagnosed and treated between 2002 and 2010) and the target population (patients diagnosed between 2018 and 2022) are completely non-overlapping, this represents a transportability problem. Commented versions of the analytic and cohort creation code are available upon request.

### Harmonizing Covariates

First, we harmonized covariates between the two populations. Age in the trial was recorded as membership in one of six age groups at baseline and race categories included in the trial were less granular than those available within the NCDB data. The trial also recorded two key potential modifiers (estrogen and progesterone receptor status) as either “negative” or “positive/unknown”, while NCDB includes detailed information on receptor status. Similarly, because testing for human epidermal growth factor receptor 2 (HER2) was less common in routine oncology practice in the early 2000 s [50], 53% of patients had unknown HER2 status in the trial vs. only 10% in NCDB. This led us to collapse HER2 status into either “positive” or “negative/unknown” for trial and target population participants (as negative cases in SEER make up the majority of unknowns) [51].

Table 2 shows the distributions of harmonized covariates in the forms used for IOW and outcome modeling in both the trial and NCDB populations, as well as the distribution of covariates after weighting. Note that the large differences in characteristics like age and HER2 status resulted in limited ability to balance other covariates and, in some cases, the IOW-weighted trial proportions not falling between the trial and NCDB patients. To minimize violations of the conditional exchangeability assumption for the target population ATE (i.e., for internal and external validity), we included all variables associated with the outcome that were measured in both populations. However, this approach can increase the likelihood of violations of the positivity assumption if certain variable combinations are rare.

### Weighting

Weights for the AC and T arms were estimated separately to ensure both achieved balance on key covariates with the trial. This approach is equivalent to fitting inverse probability of treatment weights in the trial to achieve internal conditional exchangeability and then overall IOW to achieve external conditional exchangeability assuming sufficient sample size and correct model specification. To do this, we split the trial into two datasets (one for each arm), and then “stacked” each arm with the NCDB data. Within these two datasets, we fit multivariable logistic regression models predicting the probability of being a trial participant based on race, ethnicity, tumor size, number of positive lymph nodes, type of breast surgery, age group, hormone receptor status, and HER2 status. We then assigned each trial participant a weight equal to their inverse covariate-conditional odds of study membership.

**Fig. 3:**
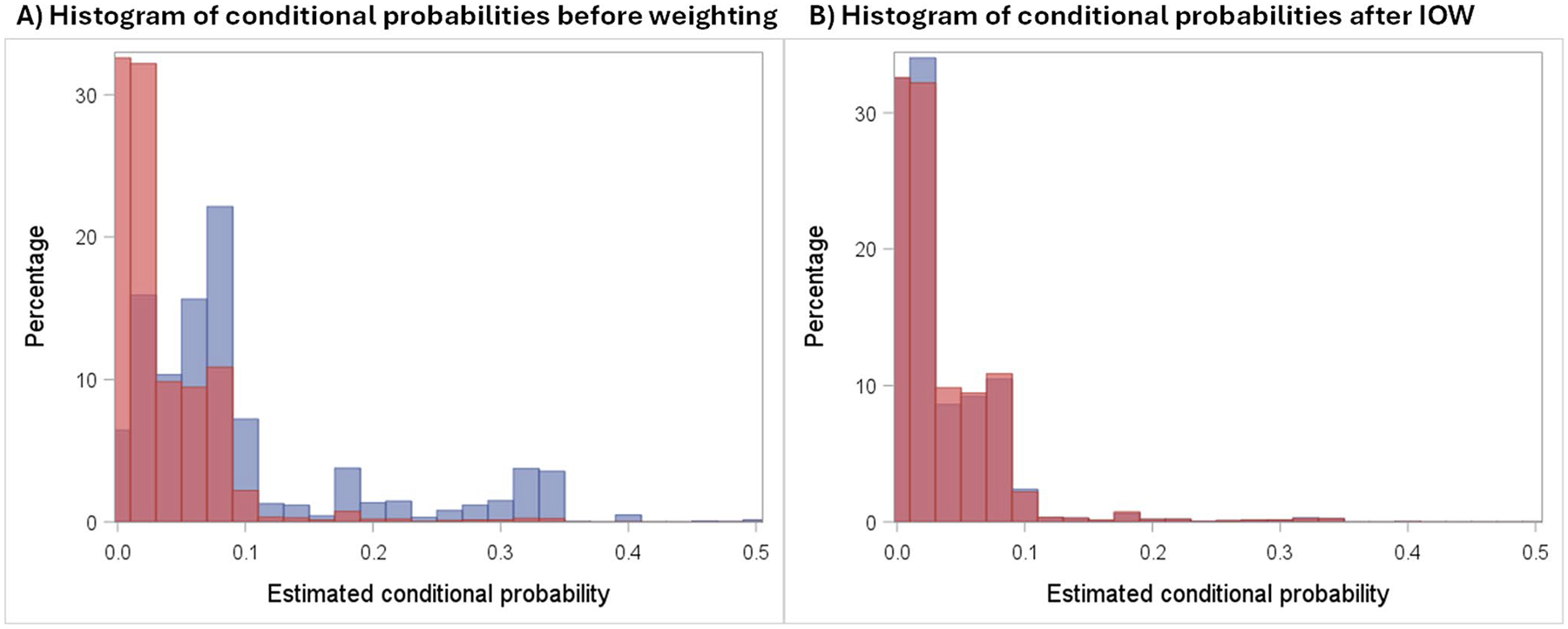
Histograms illustrating the distribution of probabilities estimated from the multivariable logistic regression model before (panel **A**) and after (panel **B**) applying inverse odds weights to the trial participants. The red columns represent the NCDB target populations while the blue columns represent the trial participants

The initial multivariable logistic regression model only included main effect terms and failed to properly balance covariates (particularly age group) between the weighted trial and NCDB data (absolute standard mean differences > 0.100), so we added interaction terms between age category and the other covariates. After weights balanced covariates satisfactorily, we calculated the 6-year difference in the risk of all-cause mortality in the AC and T arms using a weighted Kaplan-Meier estimator and obtained the confidence limits from the 2.5th and 97.5th percentiles of 2,000 bootstrap iterations resampling both the trial and target populations. Figure 3 shows the distribution of predicted probabilities of sampling comparing the trial and target before and after IOW. As discussed earlier, external positivity only requires counterparts for the target participants among the trial participants, not vice-versa, meaning that the lack of NCDB patients with predicted probabilities greater than 0.3 is not a threat to external validity.

### Outcome Modeling with Standardization

**Fig. 4:**
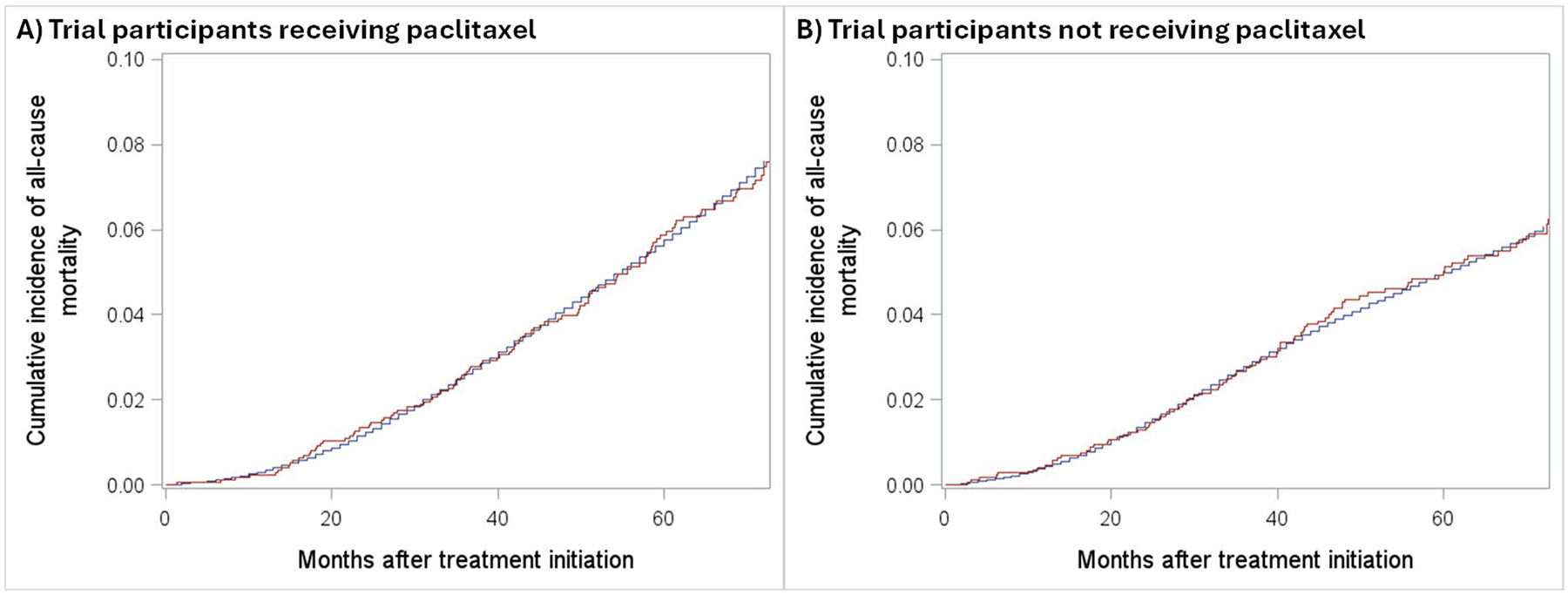
Cumulative incidence curves contrasting predicted (blue) and observed (red) risk of all-cause mortality during the first 6 years following treatment among patients in the paclitaxel arm of the trial (panel **A**) and the AC arm of the trial (panel **B**)

Due to the long follow-up duration and considerable loss to follow-up, we did not directly model the 6-year risk of death. Instead, we divided follow-up into monthly intervals and fit separate pooled multivariable logistic regression models in each arm predicting outcomes in trial participants [52]. These models included the same covariates as the weighting model and included a linear, quadratic, and cubic term for months since baseline to account for variation over time. To check that the predictions from the model aligned with the observed outcomes (i.e., the “natural course”) in the trial, we compared the actual probability of death over the course of the first 6 years of follow-up using a Kaplan-Meier estimator among trial participants vs. model predictions (see Fig. 4) [53]. After we were satisfied with the models’ performance, we applied them to the NCDB data to predict 6-year risks of all-cause mortality in the target population for each arm. The ATE was estimated as the difference in predicted outcomes between arms with confidence limits obtained from the 2.5th and 97.5th percentiles of 2,000 bootstrap iterations, resampling both trial and target populations.

## Results

Patients in the target population were older (proportion over 60 in the trial: 25.6%, proportion over 60 in NCDB: 60.1%), less likely to have 0 positive lymph nodes (proportion with 0 positive lymph nodes in the trial: 90.5%, proportion in the target: 82.6%), and less likely to have HER2 negative cancer (proportion HER2 negative in the trial: 32.2%, proportion in the target: 10.8%). The difference in 6-year risks of all-cause mortality in the trial comparing T to AC was 1.3% (95% CI: −0.5%, 3.2%). After weighting to NCDB data, T arm risk was similar (7.3% originally vs. 7.2% after weighting) and AC arm risk was lower (5.9% originally vs. 4.7% after weighting), resulting in a greater risk difference in the NCDB target (2.4%; 95% CI: −0.3%, 5.5%) than the original trial. Outcome modeling suggested a higher T arm risk and a lower AC arm risk (predicted risk in the T arm: 7.8%, predicted risk in the AC arm: 5.3%, difference in all-cause mortality = 2.6%, 95% CI: −0.1%, 5.4%). As expected given its fully parametric nature, outcome modeling was slightly more precise than weighting (confidence limit differences of 5.6% and 5.8%, respectively).

## Conclusion

Transporting effect estimates from studies (especially randomized trials with highly selected study samples) to external target populations is increasingly recognized as an important element of epidemiologic research. Providers and stakeholders are more interested than ever in whether study results apply to the kinds of patients they actually see. Greater availability of data on study populations and potential target populations with clinical and public health importance [54]has also led to a rise in opportunities to use quantitative methods to transport effect estimates [55]. While these methods may seem intimidating at first, they share much in common with methods for addressing threats to internal validity and are straightforward to implement in existing statistical software.

That is not to say that everything is completely settled and there is nothing left to discover about transportability, generalizability, external validity, and selection bias, however. Formal definitions of these and related terms are constantly changing and shifting to adapt to new discoveries and theoretical frameworks. Some external validity issues (e.g., extending inferences from a study sample in Georgia to a target population in the wider United States) may be called generalizability questions by some and transportability questions by others. Additionally, ongoing work in the field includes, but is not limited to, comparing the performance of different analytic methods, exploring ways to perform sensitivity analyses that account for unmeasured outcome predictors and effect measure modifiers [56, 57], identifying novel applications for these methods in meta-analytic and subgroup contexts [58, 59], and methods for “fusing” transported estimates with those generated from the target population [60, 61]. As these methods are applied more and more frequently to real-world substantive scenarios, further areas for innovation are bound to be identified.

Ultimately, transporting transforms “what if” questions about effects within study populations into “what if” questions about effects in new (and potentially more clinically relevant) target populations. This transformation is not without risks, however. In addition to requiring additional analyses, transporting requires conditions above and beyond those needed to achieve internally valid estimates in study populations. In the end, though, the potential payoffs for achieving this transformation and answering “what if” questions about populations that we can intervene on, rather than “what if” questions about populations that we already have measured outcomes in, can be large enough to offset the risks.

## Figures and Tables

**Table 1:** Strengths of different methods for transporting effect estimates

Method	Strengths
Inverse odds weighting (IOW)	-Easily combined with weights to address other biases (e.g., censoring weights or treatment weights) -Can inspect size + distribution of weights as diagnostics -Can evaluate performance via standardized mean differences -Final analysis is just a weighted version of the original analysis
Outcome modeling	-Will usually be more precise than weighting in simple contexts with minimal censoring -Can check performance of outcome models against the natural course to evaluate model misspecification -Built-in ability to extrapolate allows for contrasting regimens that were rare or unobserved in the source population
Augmented IOW	-Will generate unbiased estimates if either the sampling and censoring or outcome models are correctly specified -Will increase precision vs. convention inverse odds weighting -Can accommodate censoring with some additional steps
Targeted maximum likelihood estimation (TMLE)	-Will never give estimates outside the bounds of the effect measure -Can incorporate multiple machine learning methods via SuperLearner in TMLE R packages

**Table 2:** Characteristics of trial participants, the national cancer database patient target population, and the trial participants after inverse odds weighting (IOW)

Covariate	Trial *N* (%)	NCDB patient *N* (%)	IOW trial *N* (%)
Total N	3,487	46,739	95,210.5
Race *N* (%)			
Asian	62 (1.8%)	2,264 (4.8%)	4,971.6 (5.2%)
Black	380 (10.9%)	5,372 (11.5%)	12,248.0 (12.9%)
Native American or Pacific Islander	39 (1.1%)	243 (0.5%)	416.2 (0.4%)
White	3,006 (86.2%)	38,860 (83.1%)	77,574.7 (81.5%)
Ethnicity *N* (%)			
Non-Hispanic	3,329 (95.5%)	44,224 (94.6%)	89,349.3 (93.8%)
Hispanic	158 (4.5%)	2,515 (5.4%)	5,861.2 (6.2%)
Tumor size *N* (%)			
Less than 2 cm	2,259 (64.8%)	31,387 (67.2%)	63,158.5 (66.3%)
≥ 2 cm	1,228 (35.2%)	15,352 (32.9%)	32,052 (33.7)
Number of positive lymph nodes *N* (%)			
0	3,157 (90.5%)	38,587 (82.6%)	74,116 (77.8%)
1	231 (6.6%)	5,492 (11.8%)	13,178.0 (13.8%)
2	74 (2.1%)	1,837 (3.9%)	5,176.7 (5.4%)
3	25 (0.7%)	823 (1.8%)	2,739.8 (2.9%)
Type of mastectomy *N* (%)			
Partial	2,262 (64.9%)	29,224 (62.5%)	58,517.7 (61.5%)
Full	1,225 (35.1%)	17,515 (37.5%)	36,692.8 (38.5%)
Age group *N* (%)			
20–39	262 (7.5%)	1,475 (3.2%)	3,177.1 (3.3%)
40–59	2,333 (66.9%)	17,161 (36.7%)	35,849.8 (37.7%)
60+	892 (25.6%)	28,103 (60.1%)	56,183.5 (59.0%)
HER2 positivity *N* (%)			
Positive	256 (7.3%)	4,472 (9.6%)	9,613.0 (10.1%)
Negative/unknown	3,231 (92.7%)	42,267 (90.4%)	85,597.5 (89.9%)
ER/PR receptor status *N* (%)			
Receptor negative	1,123 (32.2%)	5,040 (10.8%)	10,292.3 (10.8%)
Receptor positive/unknown	2,364 (67.8%)	41,699 (89.2%)	84,918.1 (89.2%)

## Data Availability

Data from the National Cancer Database is not publicly available but is available at participating institutions. Code to create the cohort is available upon request. Data from Project Datasphere can be requested via their online portal.
